# Prevalence, incidence and mortality of diabetes mellitus in adults in Germany – A review in the framework of the Diabetes Surveillance

**DOI:** 10.17886/RKI-GBE-2017-062

**Published:** 2017-10-09

**Authors:** Christin Heidemann, Christa Scheidt-Nave

**Affiliations:** Robert Koch Institute, Department of Epidemiology and Health Monitoring, Berlin

**Keywords:** DIABETES MELLITUS, PREVALENCE, INCIDENCE, MORTALITY, EPIDEMIOLOGY

## Abstract

Continuous monitoring of the key epidemiological indicators of diabetes is necessary for evaluating the magnitude of diabetes as a public health problem, but is currently not being undertaken in Germany. A comprehensive literature review covering the last decades was conducted to give an overview of population-based studies reporting on diabetes prevalence, diabetes incidence, and diabetes-related mortality among adults in Germany. This review differentiates between known and unknown diabetes, but not between individual types of diabetes.

Numerous studies have identified a considerable increase in the prevalence of known diabetes among the adult population over time. Until the 1960s, the prevalence of known diabetes remained below 1%. However, current nationwide estimates for Germany are much higher and range between 7.2% (population aged 18 to 79 years) based on health examination surveys of the Robert Koch Institute (RKI), 8.9% (population aged 18 years and over) based on RKI telephone health interview surveys and 9.9% (among all age groups) based on statutory health insurance data. Few available estimates point to an increase in the incidence of known diabetes since the 1960s. For example, a comparison of data from the diabetes register of the former German Democratic Republic (GDR) in 1960 with current follow-up data from RKI survey participants shows that incidence rates increased from 1.2 (all age groups) to 6.9 (population aged 18 to 79 years) per 1,000 person-years. Data on diabetes-related mortality are also scarce, but indicate that excess mortality persists among people with known diabetes compared to those in the same age group without the condition, despite the finding of decreasing mortality rates among people with known diabetes. For example, the mortality rate based on early data from the GDR diabetes register was 1.9-fold higher among people with known diabetes than among the general population; current mortality follow-up data of RKI survey participants show a 1.7-fold higher mortality rate among people with known diabetes compared to those without the condition. Given the limited data that are currently available and the considerable variation of diagnostic criteria, it is not possible to estimate time trends in the prevalence, incidence or mortality of unknown diabetes.

An extension of available health monitoring approaches and an improved use of existing data sources for secondary analysis are needed for a reliable evaluation of dynamics in diabetes epidemiology in Germany. To achieve these goals, a national diabetes surveillance system is currently being established under the auspices of the RKI.

## 1. Introduction

**Info box 1: Prevalence [66, 67]**

The frequency of a specific disease among a population at a particular time. It is usually expressed as a percentage (proportion) of a given population.

Diabetes mellitus is a metabolic disorder involving a disruption of the regulation of blood glucose levels [1]. It results in chronically elevated blood glucose concentrations, which, if left untreated or treated insufficiently, can lead to serious complications including myocardial infarction, stroke, renal failure, blindness and amputations. Clearly, it can therefore reduce people’s quality of life and life expectancy, while also producing high levels of costs for health care systems [2].

Information about the spread of diabetes mellitus (Prevalence, Info box 1) is particularly relevant to attempts to classify the disorder within the public health context. Around 3,500 years ago, descriptions of symptomatology demonstrate that severe cases of diabetes were rare [3].Even as late as the first half of the 20th century, the prevalence of diabetes in Europe was still estimated to be considerably lower than 1% [4, 5]. However, since the 1960s, there has been a marked increase in the prevalence of diabetes in Germany that has led it to be viewed as endemic [6]. In fact, an alarming increase in the prevalence of diabetes has occurred throughout the world [7]; so much so that this situation has been described as a ‘diabetes pandemic’ [8, 9]. In addition to known (medically diagnosed) diabetes, unknown (medically undiagnosed) diabetes also plays an important role because it is suggested that a large number of cases go unreported [10]. There are estimations that point to a period of latency between the onset of diabetes and a medical diagnosis of the condition of at least six years on average [11]. During this time, a considerable proportion of people with unknown diabetes begin to develop diabetes-specific complications [12-14]. However, changes to the criteria used for diagnosis (Info box 4) and diabetes screening could lead to a shift in the ratio of unknown to known cases over time.

Time trends in diabetes prevalence are directly associated with developments in the rate of new cases (Incidence rate, Info box 2) and the death rate (Mortality rates, Info box 3) within a given population [15]. In turn, the incidence rate is closely associated with changes in behaviour (such as dietary habits, physical activity and associated body weight) as well as living conditions (such as economic, social and environmental factors at the individual and regional level) that have an impact on diabetes development. Apart from increases in life expectancy in the general population, the mortality rate among people with diabetes is particularly influenced by the quality of diabetes care. In addition, demographic changes (such as population ageing and migration) play a role in epidemiological developments linked to diabetes. In Germany – as in most other countries – information about the interplay of the prevalence, incidence and mortality rates linked to diabetes is limited due to the lack of continuous data collection [16-18].

This article aims to summarise available data on the prevalence, incidence and mortality of diabetes among adults in Germany and to describe time trends wherever possible. It considers both known and unknown diabetes. This article also explores approaches that could be used to continuously monitor key indicators of dynamics in diabetes epidemiology in Germany.

**Info box 2: Incidence [66, 67]**

The frequency of new cases among a population within a given time period. It is often expressed as a percentage (proportion) of new cases within a population (cumulative incidence) or the number of new cases per 1,000 person-years (incidence rate).

*Cumulative incidence (%):* The number of new cases related to the number of people at risk; in other words, the percentage of a population that does not have the disease in question at the beginning of a defined period (for example a ten-year study period) but that could develop the disease during this time. As an example, people who already have diabetes at the start of a study period are excluded from calculations of cumulative incidence.

*Incidence rate (per 1,000 person-years):* The number of new cases related to the person-time at risk; in other words, the number of new cases related to the time span accumulated by all of the people who are at risk of developing the disease and among whom it could possibly be observed during the study period. As an example, not everyone is at risk of getting diabetes during the entire study period because they may either be diagnosed with diabetes or die from other causes before the study has been completed.

## 2. Method

A narrative literature review of the PubMed bibliographic database was conducted to identify studies that have published data on diabetes prevalence, incidence and mortality in Germany. In addition, we hand-searched the bibliographies of relevant original research articles and literature reviews. However, only studies that directly provided or permitted calculation of the following data on prevalence, incidence or mortality were included within this review: prevalence as a percentage of the population with diabetes (Info box 1); incidence as a rate, in other words, as the number of new cases of diabetes per 1,000 person-years (Info box 2); age-standardised or age-adjusted mortality rate comparing all-cause mortality rates among people with diabetes to rates among people without diabetes or in the general population (Info box 3). Given the limited availability of data on incidence, studies were also included if they provided current nationwide estimates of the cumulative incidence (Info box 2). However, studies that only provided data on children or adolescents, or on population subgroups at particular risk of diabetes (such as people with obesity, a history of heart disease or those living in nursing homes), were not included in the review. Depending on the study in question, ‘diabetes’ was usually defined as all types of the disorder or just type 2 diabetes – the most predominant form of diabetes in adults [10, 18]. Detailed descriptions of the study populations and the definition of diabetes used in the studies included in this review are set out in the figures and tables presented below.

## 3. Prevalence

### 3.1 Prevalence of known diabetes

**Figure 1:**
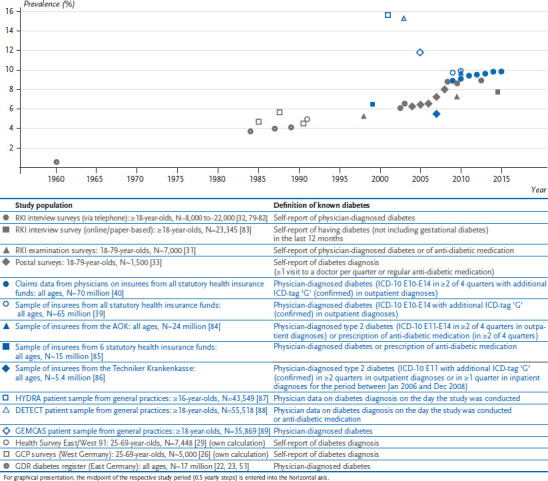
Nationwide studies providing data on the prevalence of known diabetes among adults in Germany

**Figure 2:**
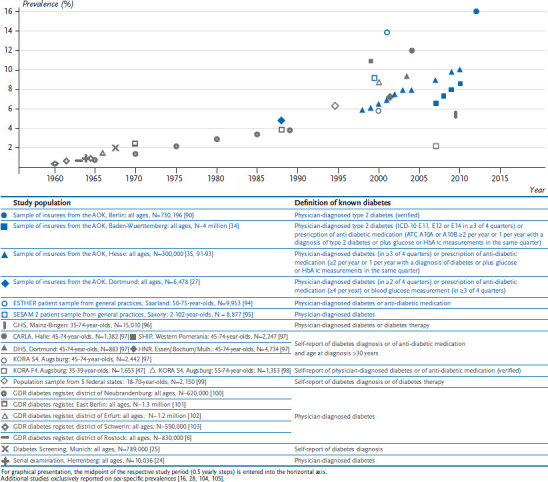
Regional studies providing data on the prevalence of known diabetes among adults in Germany

Numerous estimates of the prevalence of known diabetes are available from various studies that have been conducted over recent decades. The individual estimates of prevalence from studies undertaken after around 1960 are summarised in Figure 1 (for national-level studies) and in Figure 2 (for regional studies). Overall, the available data demonstrate that the prevalence of known diabetes has strongly increased over time.

Until the beginning of the 20th century, prevalence estimates of known diabetes were based on mortality and clinical case statistics; these identified a prevalence of between 0.2% and 0.4% [4, 5, 19]. Estimates made during the Second World War, which were derived from statistics covering the provision of insulin and dietary supplements to diabetes patients, suggest a decrease in prevalence. In part, this is due to the increased mortality among people with diabetes due to deficient or low-quality medication and food supplies as well as a higher susceptibility to infection [5, 19].

Living conditions improved in the 1950s and 1960s. This went along with an increased intake of high-calorie foods, reduced levels of physical activity and increases in the prevalence of overweight and obesity in the population; at the same time, life expectancy among people with diabetes increased due to improved treatment. As a result, the prevalence of known diabetes increased considerably [5, 6, 20-22]. In addition, diabetes screening activities mainly conducted in East Germany (the former German Democratic Republic, GDR) and to a lesser degree in West Germany had a role in increasing the prevalence of known diabetes due to better detection of undiagnosed diabetes [4, 6, 20, 22, 23]. Data from the GDR diabetes register, which covers almost all diabetes cases treated in the country between 1960 and 1989, show a continuous increase in prevalence during this period from 0.6% to 4.1% [23]. While there is no comparable database to describe time trends for West Germany during this time period, estimates that are available from various sources suggest that the prevalence in West Germany increased by a similar magnitude [24-27].

**Info box 3: Mortality [66, 67]**

The frequency of deaths among a population within a given time period. This is often provided as a percentage (proportion) of deaths within a population (cumulative mortality) or the number of deaths per 1,000 person-years (mortality rate).

*Age-standardised or age-adjusted mortality rates:*

Age-standardisation or age-adjustment is used to compare the rate of death among population groups with different age structures. These statistical methods can provide an assessment of a mortality rate that is independent of demographic differences. As an example, in this article age-standardised or age-adjusted mortality rates are compared between people with diabetes and the general population or people without diabetes. The resulting higher risk of death (known as the standardised mortality ratio or hazard ratio, Table 3) is referred to here as the excess mortality of people with diabetes compared to the reference group.

From 1990 until about 2000, data available from population studies offer no evidence of a further rise in the prevalence of known diabetes. Population-based surveys conducted in the Augsburg region (Cooperative Health Research in the Region of Augsburg, KORA; Monitoring Trends and Determinants in Cardiovascular Disease, MONICA) between 1989/1990 and 1999-2001 [28] and a comparison of data from the German nationwide survey (Nationwide Health Survey, NUS) conducted between 1990 and 1992 with data from the German National Health Interview and Examination Survey 1998 (GNHIES98) conducted between 1997 to 1999 [29] do not demonstrate an increased prevalence. Moreover, even after comparisons over time were expanded to include data from the RKI telephone health interview surveys (GSTel) conducted between 2002 and 2005, no increase over time was observed [30].

During the first decade of the 21st century, data from periodically repeated nationwide examination, telephone and postal surveys [31-33], as well as trend analyses based on insurance data from AOK Baden-Württemberg and AOK Hesse [34, 35], all demonstrate a clear increase in prevalence. According to data from the RKI examination surveys conducted between 1997 and 1999 (GNHIES98) and 2008 and 2011 (German Health Interview and Examination Survey for Adults, DEGS1), the prevalence of known diabetes rose from 5.2% to 7.2% among persons aged 18 to 79 years [31]. Health insurance data covering everyone insured by AOK Hesse between 2000 and 2009 showed a rise from 6.5% to 9.7% [35]. Differences in prevalence estimates derived from these and other studies conducted over a similar time period (Figures 1 and 2) are most likely attributable to differences in criteria used to define diabetes and in the groups of people included in the studies in question, which can differ according to the data source (Info box 5). Consistent across studies based on survey and health insurance data, about one third of the observed increase is attributable to demographic ageing [31, 35]. Further reasons for the current increase in prevalence may be improvements in early disease detection (such as increased awareness among doctors or changes in diagnostic criteria: see Info box 4), partial improvements made to diabetes care (such as the introduction of Disease Management Programmes) [36, 37] and the associated longer life expectancy. In addition, changes in the prevalence of behavioural risk factors also need to be considered. However, these demonstrate partly opposing trends and – according to a summary measure provided by the German Diabetes Risk Score – provide no evidence of a current increase in the overall level of risk [38].

Establishing a continuous monitoring system for the prevalence of known diabetes among adults in Germany appears feasible. Time trend analyses need to consider continuously collected data from nationwide, population-based RKI interview and examination surveys [31, 32] as well as routine data for secondary analysis available at the national level within the statutory health insurance system(Info box 5) [39, 40]. A comprehensive analysis is essential in this context, since the available data sources all have specific strengths and limitations (Info box 5).

**Info box 4: Laboratory criteria for the diagnosis of diabetes over time**

**Table:** 

	Fasting glucose^1^	1h-OGTT glucose^2^	2h-OGTT glucose^2^	HbA1c^3^
WHO 1965 [68]	–	–	≥7,2mmol/l (≥130mg/dl)	–
NDDG 1979 [69]	≥7,8mmol/l (≥140mg/dl)	≥11,1mmol/l (≥200mg/dl)	≥11,1mmol/l (≥200mg/dl)	
WHO 1980 [70]	≥8,0mmol (≥145mg/dl)	–	≥11,0mmol/l (≥198mg/dl)	
WHO 1985 [71]	≥7,8mmol/l (≥140mg/dl)		≥11,1mmol/l (≥200mg/dl)	
ADA 1997 [72]	≥7,0mmol/l (≥126mg/dl)			
WHO 1999 [73]				
ADA 2010 [74]				≥48mmol/mol (≥6,5%)
DDG 2010 [75]				
WHO 2011 [76]				

Abbreviations: ADA: American Diabetes Association, DDG: German Diabetes Association, NDDG: National Diabetes Data Group, WHO: World Health Organization

1. Fasting glucose: Glucose measured after a period of fasting that lasts for at least 8 hours or at least 10 hours/overnight depending on the guideline in question. Measurements are made using venous plasma.
2. OGTT glucose: Glucose measured in the oral glucose tolerance test (OGTT), i.e. 2 hours (or 1 hour according to earlier guidelines [69]) after drinking a solution of 75g glucose (or 50g/100g glucose according to earlier guidelines [68]) after a period of fasting. Measurements are made using venous plasma.
3. HbA1c: Glycated haemoglobin, i.e. form (A1) of haemoglobin to which the glucose links to (glycation). The proportion of HbA1c compared to the total level of haemoglobin represents the average glucose concentration over the past few weeks. Measurements are made using whole blood.

Some guidelines also refer to measurements of random glucose for the diagnosis of diabetes (i.e. glucose measured at any time of the day, regardless of the time since the last food intake) using ≥11.1 mmol/l (≥200mg/dl) as a cut-off in the presence of classic symptoms of diabetes (unexplained weight loss, excessive urine excretion, excessive thirst).

For further information on laboratory methods, requirements for measurement and repeated testing, please refer to the detailed descriptions provided in the references. The same applies to diagnostic criteria based on glucose measurements in capillary or whole blood as well as for the criteria used to diagnose gestational diabetes.

### 3.2 Prevalence of unknown diabetes

Some studies have been conducted over recent decades on unknown diabetes; Table 1 summarises the prevalence estimates that they have identified. A number of major systematic diabetes screenings and serial examinations that were conducted during the 1960s are included as examples. Numerous other screening activities have been summarised elsewhere [4, 19, 20, 41]. In general, the data on unknown diabetes is fragmented and a reliable analysis of trends is not feasible due to the varying criteria used to define the condition.

The earliest estimates of the prevalence of unknown diabetes are based on screenings undertaken during the 1950s and 1960s, which were mainly based on urine glucose screening (glucosuria screening) in combination with heterogeneous forms of follow-up examinations. These earlier estimates usually suggest a prevalence of below 1% or a ratio of persons with known diabetes to newly diagnosed cases of about 1:1 [42]. As of the 1970s, glucosuria screening, which has a low sensitivity, moderate specificity and an unfavourable cost-benefit ratio, became increasingly less important [23, 43, 44].

Subsequent estimates of prevalence start to become available during the mid-1990s. These are mainly derived from regional cohort studies and are partly based on fasting blood glucose levels in combination with glucose values measured 2 hours after an oral glucose tolerance test (2h-OGTT glucose) or at a random time (random glucose) (Info box 4). However, some are based on measurements of glycated haemoglobin (HbA1c). This method is now recognised as a criterion for diagnosis (Info box 4) and it is especially employed in epidemiological studies because HbA1c measures are not affected by fasting time. Nevertheless, as the studies employed different methods, and each method relates to a different aspect of glucose metabolism [45], they also identified different groups of people. Therefore, study results differ considerably depending on the method employed by the study in question [46]. Different study regions or age ranges within individual study populations makes a direct comparison of prevalence even more difficult. For example, the KORA F4 study that covers the Augsburg region employed fasting glucose levels and 2h-OGTT glucose measurements and identified a prevalence of unknown diabetes of 2.0% among 35- to 59-year-olds and of 3.9% among 35- to 79-year-olds between 2006 and 2008 [47, 48]. Using the same criteria the Study of Health in Pomerania (SHIP)-TREND, which covers Western Pomerania and was conducted between 2008 and 2012, found a prevalence of 7.1% among 35- to 79-year-olds [48]. Data from nationwide RKI health examination surveys that are based on HbA1c measurements identified a 3.4% prevalence of unknown diabetes between 1997 and 1999 and a 2.0% prevalence between 2008 and 2011 among 18- to 79-year-olds [49]. This study, which is still the only one to have employed a comparable definition of unknown diabetes at two points in time, identified a decrease in the prevalence of unknown diabetes over the last decade.

**Info box 5: Primary and secondary data**

*Definition:* In contrast to primary data, secondary data are data that are not directly collected for a research interest that was specified in advance or that are evaluated differently from their intended usage [64].

*Data sources:* Primary data sources that are important for the identification of the frequency of diseases include 1) the examination and interview surveys conducted regularly at nationwide level by the Robert Koch Institute (RKI) [77] and 2) ongoing cohort studies such as the GNC that is being undertaken in 18 study centres [50]. Secondary data sources include administrative data routinely collected within the German social security and health system for documentation and reimbursement. Of particular importance in this context are nationwide routine data that come from multiple statutory health insurers such as 1) the data reported to the German Federal Insurance Office (BVA) for the Morbidity-oriented Risk Structure Compensation (Morbi-RSA). Since 2014, these data have been merged for research purposes in accordance with the Data Transparency Regulation (DaTraV) and are held by the German Institute of Medical Documentation and Information (DIMDI). Another important source of nationwide routine data is 2) the data collected on people with statutory health insurance sent for billing purposes by contract doctors and that are regularly analysed by the Central Research Institute of Ambulatory Health Care in Germany (Zi) [65].

*Advantages and limitations:* Primary data sources such as the RKI examination surveys often include information on health-related behaviours and laboratory measures. This permits monitoring of risk factor profiles and undiagnosed conditions, such as unknown diabetes. However, these surveys miss certain groups of people (e.g. nursing home residents, people who are very old) and not everyone who is invited actually participates (e.g. there is a lower probability of participation among people with multimorbidity). Existing data sources available for secondary analysis, such as routine data within the statutory health insurance system, in contrast, include all age groups and large sample sizes, and hence permit the conduction of stratified analyses (such as by region) as well as more timely estimates of health indicators. Nevertheless, even these data do not cover the entire population (e.g. people with private health insurance or people who do not use the healthcare system are not included) [65, 78].

In order to continuously monitor the prevalence of unknown diabetes in the adult population in Germany, it is essential that studies employ a definition that is comparable over time. Currently, this can only be done by continuing the RKI health examination surveys, which are conducted at relatively wide intervals. Nevertheless, cohort studies, such as the German National Cohort (GNC) [50], which is being conducted in 18 study centres, will also provide valuable point estimates of the ratio of people with known and unknown diabetes.

## 4. Incidence

### 4.1 Incidence rate of known diabetes

Few estimates of incidence rates (Info box 2) are available for known diabetes from studies that were conducted over the last few decades with various designs; the results are summarised in Table 2. Overall, these estimates indicate a clear increase in the incidence rate of known diabetes since 1960.

An incidence rate of 1.2 per 1,000 person-years (py) was observed from data sourced from the GDR diabetes register for 1960 [23]. Until 1989, when the registry was closed, an increased incidence rate of 3.8 per 1,000 py was observed [22, 51]. Apart from changes in people’s behaviour, the frequency of glucosuria screening activities [22] as well as changes to the diagnostic criteria used to define diabetes (Info box 4) most likely contributed to what was described as a stepwise increase in incidence rates.

For the subsequent period, point estimates from regional cohort studies indicate continued increase in diabetes incidence rates [52-54]. A recent investigation based on pooled data from five regional cohort studies (Diabetes-Collaborative Research of Epidemiologic Studies, DIAB-CORE; follow-up between 1997 to 2010) found an incidence rate of 11.8 per 1,000 py among 45- to 74-year-olds [54].

Our own analyses of nationwide data from the panel of adults who participated in two subsequent RKI health examination surveys with an average follow-up time of 12 years (follow-up period: 1997-1999 to 2008-2011) revealed an incidence rate of known diabetes of 6.9 per 1,000 py among people aged 18 to 79 years at baseline and 11.4 per 1,000 py among people aged 45 to 79 years at baseline. Based on current population estimates [55] this amounts to an estimate of about 442,000 new cases of known diabetes occuring annually among 18-to 79-year-olds in Germany. Based on routine data that are made available for research in accordance with the Data Transparency Regulation (Info box 5), a recent nationwide study has provided estimates of the type 2 diabetes incidence rate among persons 40 years and older within the German statutory health insurance system. Incidence rates amounted to 13 per 1,000 py among women and 16 per 1,000 py among men. These rates were calculated using a differential equation that took the following variables into account: 1) the prevalence of known diabetes among people with statutory health insurance between 2009 and 2010, 2) mortality rates for the general population in Germany as obtained from official statistics, and 3) the ratio of mortality rates people with and without diabetes based on estimates available from the neighbouring country of Denmark [39]. A further nationwide analysis of routine data available within the German statutary health insurance system was carried out by the Central Research Institute of Ambulatory Health Care in Germany (Info box 5;). Among persons 40 years and older, these authors found a slight decrease in the cumulative incidence (Info box 2) of type 2 diabetes from 1.63% in 2012 to 1.47% in 2014. Calculations of the proportion of new cases within a given year were based on the requirement of a three-year pre-observation period during which the participants had received no medical diagnosis of diabetes [40].

Continuous monitoring of incidence rates of known diabetes among the general adult population in Germany at the national level, such as through continued follow-up of RKI health survey participants, is currently not being realised. However, using available routine data provides a feasible approach to obtain estimates of the cumulative incidence on a regular basis [40]. It would also be possible to use available data for continous calculations of incidence rates using the known mathematical relationships between prevalence, incidence and mortality [39]. Country-wide estimates on the prevalence of known diabetes are available on a regular basis using data collected within the RKI Health Monitoring framework and from the routine data sources of the statutory health insurance system. Regular estimates of the mortality rate among the general population are made available by official cause-of-death statistics. Data on the ratio of mortality rates among people with and without known diabetes, which has been ‘borrowed’ from the neighbouring country of Denmark until now, could be frequently made available also for Germany if follow-up of RKI health survey participants for vital status (mortality follow-up) could be conducted on a regular basis [56, 57].

### 4.2 Overall incidence of unknown and known diabetes overall

The incidence rate of unknown and known diabetes combined has only recently been estimated by a number of studies. However, results from these studies are difficult to compare due to differences in study design, age range and reference region (Table 2).

Based on KORA S4/F4 cohort data (follow-up period: 1999-2001 to 2006-2008), the incidence rate for known diabetes and unknown diabetes (defined using fasting blood glucose level and 2h-OGTT glucose) combined was estimated to be 15.5 per 1,000 py among 55- to 79-year-olds from the Augsburg area [58]. A comprehensive analysis of data from the SHIP cohort in Western Pomerania (follow-up period: 1997-2001 to 2003-2006; 20- to 79-year-olds) and a nationwide sample of patient data (Diabetes Cardiovascular Risk-Evaluation: Targets and Essential Data for Commitment of Treatment, DETECT; follow-up period: 2003 to 2007/2008; people aged 18 years or above) revealed an incidence rate for known and (HbA1c-defined) unknown diabetes of 14.4 per 1,000 py [59]. Our own analyses of nationwide data from adults who participated in two RKI examination surveys spaced approximately 12 years apart (follow-up period: 1997-1999 to 2008-2011) resulted in an incidence rate of known and (HbA1c-defined) unknown diabetes of 7.9 per 1,000 py among persons aged 18 to 79 years at baseline and a rate of 12.8 per 1,000 py among those aged 45 to 79 years at baseline. Based on current population statistics in Germany [55], this corresponds to approximately 507,000 new cases of diabetes per year in the population 18 to 79 years of age.

Currently available data do not permit estimation of time trends in overall diabetes incidence rates among adults in Germany. In the future, the total incidence rate could be calculated using the differential equation mentioned above [39]. For this, estimates of the prevalence of known and unknown diabetes will be available, albeit at larger intervals, from the national RKI health examination surveys [49]. Moreover, continued mortality follow-up of RKI health survey participants would permit periodically repeated estimates of mortality rates among people with and without known or unknown diabetes [56, 57]. In addition, ongoing cohort studies in Germany will continue to contribute point estimates of overall diabetes incidence.

## 5. Mortality

### 5.1 Mortality among people with known diabetes

Only a small number of studies have provided data on diabetes-related excess mortality (Table 3), in other words, the mortality rate of people with diabetes compared to the general population or people without diabetes (Info box 3). Results from these studies show that mortality rates among people with diabetes have decreased over recent decades. However, the results also suggest that mortality rates among people with diabetes remain higher than among people of the same age who do not have diabetes.

According to early estimates based on data from the GDR diabetes register, the ratio of age-standardised mortality rates among people with known diabetes compared to the general population slightly declined from 1.9 in 1961 to 1.7 in 1987, although this decrease was not statistically significant [60].

More recently, the Erfurt Male Cohort Study (ERFORT study; follow-up period: 1973-1975 to 2003) demonstrated a 1.9-fold higher risk of death from all causes among 40- to 59-year-old men with known diabetes [61], the KORA S4 study (follow-up period: 1999-2001 to 2008/2009) identified a 2.6-fold higher risk among 55-to 74-year-olds [62]; and the GNHIES98 (follow-up period: 1997-1999 to 2008-2011) found a 1.7-fold higher risk of mortality among 18- to 79-year-olds [57]. Each study compared age-adjusted mortality rates among people with known diabetes to people without known or unknown diabetes.

Official statistics on causes of death provided by the Federal Statistical Office provide data for monitoring mortality rates in the general population (of 100,000 inhabitants) [63]. However, the mortality follow-up of persons participating in the national RKI health examination surveys is currently the only nationwide data source that can be used to calculate population-based mortality rates among adults with diabetes compared to those without diabetes [56, 57]. It would therefore be important to continue the follow-up of survey participants’ vital statistics (so far running for GNHIES98 and DEGS1). Looking forward, the mortality follow-up of people participating in the on-going German National Cohort [50] as well as mortality data that will be available for secondary analysis of existing data from the statutory health insurance system will also provide information about diabetes-related excess mortality.

### 5.2 Mortality among people with unknown diabetes

The only estimates of excess mortality among people with unknown diabetes that currently exist are from the three follow-up studies mentioned in the last section (Table 3). Therefore, it is currently impossible to estimate time trends in this regard.

The ERFORT study found that the risk of death among people with unknown diabetes was 1.5 times higher compared to people without diabetes [61]. The KORA S4 study identified the rate as 2.8 times higher [62] and the GNHIES98 study found a rate that was 1.9 times higher [57]. Thus, the risk of death among people with unknown diabetes is of a similar magnitude as the risk of death observed among people with known diabetes. In contrast, the studies found no increased risk of death among people with ‘prediabetes’ [57, 62] (Table 3).

The continuation of the mortality follow-up of people participating in the national RKI health examination surveys, therefore, would also be useful in order to gain regular estimates (albeit at larger intervals) of the excess mortality linked to unknown diabetes and diabetes overall [56, 57]. In addition, following up the vital statistics of participants from ongoing cohort studies could provide point estimates of excess mortality related to unknown diabetes.

## 6. Conclusion

Population-based estimates of the prevalence, incidence and excess mortality of known and unknown diabetes are key indicators in order to conduct a reliable evaluation of developments in diabetes epidemiology. Providing regular estimates of these indicators that are comparable over time, therefore, is a major goal of the national diabetes surveillance system that is currently being established in Germany. With the exception of the prevalence of known diabetes (where regularly collected primary and secondary data demonstrate an increase over the last few decades), the data being collected on these key indicators of diabetes epidemiology in Germany is currently fragmented. Estimates of incidence rates and the excess mortality associated with known diabetes that are currently available, mainly from cohort studies, only enable cautious conclusions to be drawn on time trends. Estimates of the prevalence, incidence and mortality of unknown diabetes in Germany are scarce and do not permit the evaluation of time trends.

An expansion of existing approaches is therefore needed in order to resolve the current issues with the data. Thus, the regular continuation of the mortality follow-up of people taking part in the RKI national health examination surveys would permit monitoring of the mortality rates among people with diabetes compared to those without the condition, albeit at larger intervals. This could reduce the large gaps that exist in recurrent estimates of excess mortality in relation to both known and unknown diabetes. As demonstrated by recent studies, improved access to secondary analysis of existing data would help produce more timely estimates of the prevalence, as well as the incidence of known diabetes. Routine data available within the statutory health insurance system are of particular importance in this respect. While these routine data sources cover most of the population, certain groups of people (for example, people insured by private health insurers) are not represented in the sample. Moreover, indicators calculated based on routine data will be limited to known diabetes. The use of mathematical equations could therefore be considered as a further means of closing existing gaps in the data. As an example, population-based incidence rates of known and unknown diabetes could be derived from the mathematical relationships between the data on diabetes prevalence and excess mortality provided by the national RKI health examination surveys.

A national diabetes surveillance system is currently being established at the RKI. The various approaches and available data sources are currently being assessed with regard to their availability and whether they can be integrated into a continuous monitoring of dynamics in diabetes epidemiology as a means of providing a data-supported foundation for health policy decision-making in Germany [64, 65]. Taking into account demographic trends, a foundation could also be used for projections of burden of disease.

## Key statements

- Since the 1960s, the proportion of people with known diabetes (prevalence) has increased almost ten-fold.
- There is some evidence that the rate of people newly diagnosed with diabetes (incidence rate) has increased since the 1960s.
- Currently available data do not permit estimation of time trends in the prevalence and incidence of unknown diabetes.
- There is evidence that the risk of death among people with known diabetes is about twice as high as among people without the condition; the increased risk of death (excess mortality) among people with unknown diabetes appears to be about as high as among people with known diabetes.
- Data on key measures (core indicators) of diabetes epidemiology in Germany is yet limited, but will be expanded and consolidated within the framework of the German National Diabetes Surveillance System.

## Figures and Tables

**Table 1:** Studies providing data on the prevalence of unknown diabetes among adults in Germany

Study population	Study period	Definition of unknown diabetes	Prevalence	Reference time point*
**Nationwide surveys**				
DEGS1: 18-79-year-olds; N=7,017 [49]	2008-2011	HbA1c ≥6.5%	Total: 2.0% (Women: 1.2%; Men: 2.9%)	31 Dec 2010
GNHIES98: 18-79-year-olds; N=6,655 [49]	1997-1999	HbA1c ≥6.5%	Total: 3.8 % (Women: 3.2%; Men: 4.3%)	31 Dec 2010
			Total: 3.4 %	31 Dec 1997
GNHIES98: 18-79-year-olds; N=5,275 [29]	1997-1999	HbA1c >6.1% and either serum glucose ≥126mg/dl or glucose in urine ≥50mg/dl	Women: 2.0%; Men: 2.1%	31 Dec 1997
**Regional studies**				
KORA F4 (Augsburg): 35-79-year-olds; N=2,617;	KORA: 2006-2008	Fasting glucose ≥7.0mmol/l or 2h-OGTT glucose ≥11.1mmol/l	KORA: 3.9 %	31 Dec 2007
SHIP-TREND (Vorpommern): 35-79-year-olds; N=1,980 [48]	SHIP: 2008-2012		SHIP: 7.1%	
KORA F4 (Augsburg): 35-59-year-olds; N=1,653 [47]	2006-2008	Fasting glucose ≥7.0mmol/l or 2h-OGTT glucose ≥11.1mmol/l	Total: 2.0 % (Women: 1.6%; Men: 2.4%)	31 Dec 2007
Screening participants in routine health examinations of BASF employees: ≈16-64-year-olds; N=13,086 [106]	2004-2005	Fasting glucose ≥7.0 mmol/l or random glucose ≥11.1mmol/l	Total: 0.7%	
Screening participants of a sample of people insured by Techniker Krankenkasse (Thüringen, Düsseldorf) ≥55-year-olds; N=4,314 [107]	2003	Physician-diagnosed ‘manifest diabetes mellitus type 2’ and no self-report of diabetes diagnosis	Total: 2.8%	
HNR (Essen, Bochum, Mülheim): 45-74-year-olds; N=4,595 [108]	2000-2003	Fasting glucose ≥7.0mmol/l or random glucose ≥11.0mmol/l	Women: 3.2%; Men: 7.6%	
KORA S4 (Augsburg): 55-74-year-olds; N=1,353 [98]	1999-2001	Fasting glucose ≥7.0mmol/l or 2h-OGTT glucose ≥11.1 mmol/l	Total: 8.2% (Women: 6.9%; Men: 9.3%)	31 Dec 2000
EPIC-Potsdam: 35-59-year-olds; N=27,500 [16]	1994-1998	Fasting or random glucose	Women: 0.4%; Men: 1.0%	2007
Sample of randomly selected cities/rural districts in 5 federal states in Germany: 18-70-year-olds; N=2,150 [99]	1993-1996	HbA1c >6.0%	Total: 1.6%	
Diabetes screening programme in Munich: All ages, N=789,289 [25]	1967/1968	Urine test strip discolouration and medical confirmation in follow-up examination	Total: about 0.7-1.1%	
Diabetes screening programme of employees of administrations and of a pharmaceutical-chemical company (West Berlin) 16–65-year-olds, N=4,187 [109]	1965/1966	2h-OGTT glucose ≥7.8 mmol/l during screening and ‘manifest unknown diabetes’ in follow-up examination	Total: 1.0%	
Serial examination of the population of the town of Herrenberg: all ages, N=7,976 [24]	1964	Urine test strips with glucosuria >0.5% or urine test strips with glucosuria >0-0.5% plus medical confirmation in follow-up examination	Total: 0.6%	
Serial examination of the population in 5 areas of the district of Magdeburg: ≥14-year-olds (≥18-year-olds in one district), N=164,896 [41]	1964/1965	Abnormal result of urine glucose test and confirmation in follow-up examination	Total: 0.5%	
Serial examination of the population of the district of Schwerin: ≥14 years (1961/1962) or ≥12 years (1964/1965), N≈je 380.000 [103]	1964/1965, 1961/1962	Urine test strip discolouration and blood glucose 7.2-11.1 mmol/l 2hours after the main meal with confirmation in follow-up examination	1964/1965: 0.2% 1961/1962: 0.3%	
Serial examination of the population in 10 out of 14 areas of the districts of Neubrandenburg: 6-80-year-olds, N=318.687 [110]	1961/1962	Urine test strip discolouration and confirmation in follow-up examination	Total: 0.3%	
**Patient data**				
GEMCAS (nationwide patient sample from general practices): ≥18 years, N=35.869 (N=1.511 practices) [89]	2005	Random glucose ≥11.1 mmol/l or fasting glucose ≥7.0 mmol/l	Total: 0.9%	2003
Diabetes screening programme of the German Medical Association of the former Federal Republic of Germany (West Germany) N=1.474.827 (N=25.000 doctors) [111]	1964/1965	Urinary glucose test	Total: 1.8%	

Major systematic diabetes screening activities during the 1960s are exemplarily listed in Table 1; numerous other screenings have already been summarised elsewhere [4, 19, 20, 41]. Further studies not listed in Table 1 or Figure 1 or Figure 2 provide results on the total prevalence of known and unknown diabetes [112-114].

^*^ for age-standardisation

**Table 2:** Studies providing data on the incidence of diabetes among adults in Germany

Study population		Follow-up period*	Definition of diabetes incidence at follow-up	Incidence per 1,000 person-years	Method for consideration of bias
**Nationwide surveys**					
GNHIES98 re-participants: 18-79-year-olds, N=3.779 (own calculation)		1997-1999, 2008-2011	Self-report of physician-diagnosed diabetes or of anti-diabetic medication for the first time	**Known diabetes** 18-79-year-olds: 6.9 (Women: 7.4; Men: 6.3) 45-79-year-olds: 11.4 (Women: 10.9; Men: 12.0)	Weighting for loss of non-returnees to follow-up; standardised to population structure of Germany as of 31 Dec 1997
GNHIES98 re-participants with an examination: 18-79-year-olds, N=2.750 (own calculation)			Self-report of physician-diagnosed diabetes or of anti-diabetic medication for the first time or HbA1c ≥6.5% for the first time	**Known or unknown diabetes** 18-79-year-olds: 7.9 (Women: 9.0; Men: 6.8) 45-79-year-olds: 12.8 (Women: 12.4; Men: 13.3)	
**Register data**					
GDR diabetes register: all ages, entire population [23, 51]		Each year (reporting date 31 Dec) between 1960 and 1089	Physician-diagnosed diabetes for the first time	**Known diabetes** 1989: 3.8 1960: 1.2	
GDR diabetes register, district of Neubrandenburg: all ages, entire population [115]				**Known diabetes** 1980: 3.4 (Women: 2.2; Men: 4.5) 1976: 3.4 (Women: 2.4; Men: 4.3) 1972: 2.5 (Women: 1.9; Men: 3.4) 1970: 2.5 (Women: 2.0; Men: 3.0) 1964: 1.2 (Women: 0.9; Men: 1.5) 1960: 0.8 (Women: 0.5; Men: 1.0)	
**Regional studies**					
DIAB-CORE Consortium with SHIP (Western Pomerania), CARLA (Halle/Saale), DHS (Dortmund), HNR (Essen, Bochum, Mülheim), KORA (Augsburg): 45-74-year-olds	N=8,787 [54]	SHIP: 1997-2001, 2002-2006 CARLA: 2002-2006, 2007-2010 DHS: 2003-2004, 2006-2008 HNR: 2000-2003, 2006-2008 KORA: 1999-2001, 2006-2008	Self-report of physician-diagnosed diabetes for the first-time	**Known diabetes** Total: 11.8 SHIP: 13.0 (Women: 10.0; Men: 16.3) CARLA: 16.2 (Women: 11.7; Men: 21.9) DHS: 16.2 (Women: 15.0; Men: 17.8) HNR: 11.8 (Women: 8.6; Men: 15.3) KORA: 9.0 (Women: 7.2; Men: 11.1)	Weighting for loss of non-returnees to follow-up; standardised to population structure of Germany as of 31 Dec 2007
	N=7,250 [116]			**Known diabetes** Total: 12.6 (Women: 9.2; Men: 16.1)	
KORA S4/F4 (Augsburg): 55-74-year-olds, N=887 [58]		1999-2001, 2006-2008	Medically verified diabetes diagnosis after self-report of diabetes diagnosis for the first time or fasting glucose ≥7.0 mmol/l or 2h-OGTT glucose ≥11.1 mmol/l for the first time	**Known or unknown diabetes** Total: 15.5 (Women: 11.3; Men: 20.2)	Standardised to population structure of Germany as of 31 Dec 2007
SHIP (Western Pomerania): 20-79-year-olds, N=2,841; DETECT (nationwide sample of patients from general practices): ≥18-year-olds, N=4,936 [59]		SHIP: 1997-2001, 2003-2006 DETECT: 2003, 2007-2008	Self-report of diabetes diagnosis or of anti-diabetic medication for the first time or HbA1c ≥6.5% for the first time	**Known or unknown diabetes** Total: 14.4	
EPIC-Potsdam: 35-65-year-olds, N=27,067 [53]		1994-1998, 2005	Medically verified diabetes diagnosis after self-report of diabetes diagnosis or diabetes therapy for the first time	**Known diabetes** Total: 4.8	
MONICA Augsburg: 35-74-year-olds, N=6,166 [52]		1984-1995, 1998	Self-report of diabetes diagnosis or of anti-diabetic medication for the first time	**Known diabetes** Women: 4.0; Men: 5.8	Standardised to population structure of Germany as of 31 Dec 1989
**Health insurance data**					
Nationwide sample of insurees from all statutory health insurance funds: ≥40-year-olds [39]		2009, 2010	By differential equation calculated incidence based on the change in diabetes prevalence between 2009 and 2010 in the sample of insurees (physician-diagnosed diabetes [ICD-10 E10-E14, with the additional ICD-tag, ‘G’ (confirmed) in outpatient diagnoses]) and the mortality among people with and without diabetes in the Danish population	**Known diabetes** Women: 13; Men: 16	
Sample of insurees from the AOK Baden-Württemberg: all ages, N≈3.5 million per year [34]		2007-2009, the next year	Physician-diagnosed type 2 diabetes for the first time (ICD-10 E11, E12 or E14 in ≥3 of 4 quarters) or prescription of anti-diabetic medication for the first-time (ATC A10A or A10B ≥2 per year or 1 per year plus type 2 diabetes diagnosis or plus glucose or HbA1c measurement in the same quarter)	**Known diabetes** 2010: 8.6 (Women: 8.3; Men: 9.2) 2009: 7.7 (Women: 7.3; Men: 8.3) 2008: 8.2 (Women: 7.8; Men: 8.9)	Standardised to population structure of Baden-Württemberg as of 31 Dec of the respective year
Nationwide sample of insures from the Techniker Krankenkasse: all ages, N=5,4 million [86]		2006-2007, 2008	Inpatient type 2 diabetes diagnosis for the first time (ICD-10 E11 with additional ICD-tag ‘G’ (confirmed)) or two outpatient type 2 diabetes diagnoses in different quarters of 2008 or in/before 2008	**Known diabetes** Total: 4.1	Standardised to population structure of Germany as of 31 Dec 2008
**Patient data**					
SESAM 2 (patient sample from general practices in Saxony): 2-102-year-olds, N=8,877 (N=270 practices) [95]		10/1999-09/2000	Physician-diagnosed diabetes for the first time	**Known diabetes** Total: 3.0	

Further studies not listed in Table 2 provide cumulative incidences (percentages) [40, 84] or incidence rates (per 1,000 person-years) for subgroups of individuals with normal or impaired glucose metabolism [117].

* Baseline – follow-up

**Table 3:** Studies providing data on overall mortality among adults with diabetes compared to adults without diabetes in Germany

Study population	Follow-up period^1^	Definition of diabetes and reference group at baseline	Mortality among adults with diabetes compared to the reference group			Methods to account for bias
			Crude^2^ mortality rate^3^		Age-adjusted hazard ratio^4^	
**Nationwide surveys**						
GNHIES98 Mortality Follow-up: 18-79-year-olds, N=6,299 [57]	1997-1999, 2008-2011	**Known diabetes:** Self-report of physician-diagnosed diabetes or of anti-diabetic medication	27.4		1.7	Follow-up for vital status completed for 98%; Adjusted for age, sex
		**Unknown diabetes:** HbA1c ≥6.5%	29.4		1.9	
		**Prediabetes:** HbA1c: 5.7-5.9%/6.0-6,4%	11.3/8.6		1.0/1.0	
		Reference: No known or unknown (pre-) diabetes	4.1		1 (reference)	
**Register data**						
GDR diabetes register: all ages, All people with known diabetes (compared to the general population) [60]	Each year between 1961 and 1987	**Known diabetes:** Physician-diagnosed diabetes; Reference: general population	Non-insulin-dependent diabetes	1961: ≈47 1987: ≈67	–	Follow-up for vital status completed for nearly 100%; Standardised to age structure of the general population
			Insulin-dependent diabetes	1961: ≈64 1987: ≈77	–	
			Total diabetes	–	1961: 1.9 1987: 1.7	
**Regional studies**						
ERFORT (Erfurt area): 40-59-year-olds, N=1,125 men [61]	1973-1975, 2003	**Known diabetes:** Self-report of physician-diagnosed diabetes	10 years 20 years 30 years	21.5 38.1 43.1	2.2 2.2 1.9	Follow-up for vital status completed for 98%; Adjusted for age
		**Unknown diabetes:** 1h-OGTT glucose ≥200 mg/dl	10 years 20 years 30 years	17.0 24.5 31.5	1.8 1.5 1.5	
		Reference: No known or unknown diabetes	10 years 20 years 30 years	8.0 15.4 20.6	1 (reference)	
KORA S4 (Augsburg area): 55-74-year-olds, N=1,466 [62]	1999-2001, 2008-2009	**Known Diabetes:** Verified self-report of physician-diagnosed diabetes	30.7		2.6	Follow-up for vital status completed for 99%; Adjusted for age, sex
		**Unknown diabetes:** Fasting glucose ≥7.0 mmol/l or 2h-OGTT glucose ≥ 11.1 mmol/l	35.4		2.8	
		**Prediabetes:** Fasting glucose 6.1-6.9 mmol/l or 2h-OGTT glucose 7.8-11.0 mmol/l	13.3		1.1	
		Reference: No known or unknown (pre-) diabetes	10.5		1 (reference)	
**Patient data**						
Erfurt-Study (district of Erfurt): all ages, N=208 people with diabetes (compared to N=208 paired controls) [118]	1970, 1985	**Known diabetes:** History of physician-diagnosed diabetes of ≥20 years; Reference: paired metabolically healthy people	2.1			Follow-up for vital status completed for 93%; Case-control pairing according to age, sex, body weight

A further study, which is not listed in Table 3, exclusively provides age- and gender-specific mortality ratios [102].

^1^ Baseline – follow-up

^2^ not age-adjusted or age-standardised

^3^ per 1,000 person-years

^4^ or age-standardised mortality ratio
